# Understanding immune function as a pace of life trait requires environmental context

**DOI:** 10.1007/s00265-018-2464-z

**Published:** 2018-03-09

**Authors:** B. Irene Tieleman

**Affiliations:** Groningen Institute for Evolutionary Life Sciences, University of Groningen, 9700 CC Groningen, The Netherlands

**Keywords:** Pace of life, Immune function, Birds, Eco-immunology, Environmental adaptation

## Abstract

This article provides a brief historical perspective on the integration of physiology into the concept of the pace of life of birds, evaluates the fit of immune function into this framework, and asks what it will take to fruitfully understand immune functioning of birds in pace of life studies in the future. In the late 1970s, physiology started to seriously enter avian life history ecology, with energy as the main currency of interest, inspired by David Lack’s work in the preceding decades emphasizing how food availability explained life history variation. In an effort to understand the trade-off between survival and reproduction, and specifically the mortality costs associated with hard work, in the 1980s and 1990s, other physiological phenomena entered the realm of animal ecologists, including endocrinology, oxidative stress, and immunology. Reviewing studies thus far to evaluate the role of immune function in a life history context and particularly to address the questions whether immune function (1) consistently varies with life history variation among free-living bird species and (2) mediates life history trade-offs in experiments with free-living bird species; I conclude that, unlike energy metabolism, the immune system does not closely covary with life history among species nor mediates the classical trade-offs within individuals. Instead, I propose that understanding the tremendous immunological variation uncovered among free-living birds over the past 25 years requires a paradigm shift. The paradigm should shift from viewing immune function as a costly trait involved in life history trade-offs to explicitly including the benefits of the immune system and placing it firmly in an environmental and ecological context. A first step forward will be to quantify the immunobiotic pressures presented by diverse environmental circumstances that both shape and challenge the immune system of free-living animals. Current developments in the fields of infectious wildlife diseases and host-microbe interactions provide promising steps in this direction.

## Physiology, life history, and behavior united in the pace of life

Since the introduction of the pace of life concept at the end of the twentieth century, several research disciplines have increasingly become integrated, notably comparative physiology/ecophysiology, life history ecology, and behavioral biology. Each has its own rich body of knowledge and familiarity with these can positively contribute to the integration. For example, physiologists have a long-standing interest in connecting physiological mechanisms to demographic traits such as survival and reproduction. More than a century ago Rubner (1908), later enhanced by Pearl (1928), proposed the rate of living theory, based on the observation that the faster an organism’s metabolism, the shorter its life span. After the Second World War, comparative physiologists built upon this work on the rate of living by uncovering differences in metabolic rate among animals in different environments, laying the foundation for ecological energetics (e.g., Scholander 1950a, b; Bartholomew and Dawson 1953, Schmidt-Nielsen 1972, 1984). During these same decades, among animal ecologists, understanding reproduction and survival became major topics of research, effectively spawning the field of life history ecology and evolution (for a review see Ricklefs 2000a). More than half a century after the inception of the rate of living theory, animal ecologists fascinated by life history trade-offs started to include measurements of the underlying physiology to understand factors driving the ecology and evolution of life history phenomena such as reproduction and migration. Towards the end of the twentieth century, the concept of the pace of life emerged in the literature, basically replacing and combining the rate of living theory that had inspired comparative physiologists and life history theory that had guided animal ecologists. Also during this time, the discovery that animals have individually consistent coping styles—or personalities—with significant ecological and evolutionary consequences spawned a new field of behavioral research (e.g., Verbeek et al. 1994, 1996; Dingemanse et al. 2004). Where the comparative physiologists emphasized among-species comparisons, the animals ecologists aimed to reveal trade-offs at the within-individual level using experiments, and most recently, the behavioral biologists interested in personalities focused on understanding among-individual variation. In order to advance the integration of these various research traditions, in this article, I will use the extensive knowledge of birds to (i) provide a brief history of the integration of physiology into the avian life history ecology framework, (ii) review comparative among-species studies and experimental within-individual studies to evaluate the fit of immune function as a physiological life history trait that represents investment in self-maintenance/survival in this framework, and (iii) explore what it takes to fruitfully understand immune function in avian pace of life studies in the future.

## Life history variation and trade-offs at the species and individual level: a role for physiology

At the species level, survival and reproduction are inversely associated (Ricklefs 2000b). Early bird ecologists had different explanations for this inverse association (for historical overviews see Ricklefs 2000a, b): David Lack, who worked in temperate zone Europe with bountiful springs, proposed that food supply drove avian life history variation, through its primary effect on reproduction. Reproduction, in turn, affected mortality, through density dependence effects. Contemporary ecologists Alexander Skutch, who worked in tropical American sites, and Philip Ashmole, who studied birds on oceanic islands, proposed that the primary driver of life history variation was (extrinsic) mortality. They agreed that mortality determined population density and thereby affected reproduction either directly (Skutch) or through food availability (Ashmole). Despite their different views of the mechanisms underlying life history variation, all three of these early ecologists looked to the ecological factors characterizing the environments in which they worked to explain the inverse relationship between survival and reproduction.

At the within-individual level, the trade-off between survival and fecundity has been and continues to be heavily studied. Especially in Europe, much of the life history work on birds was built on Lack’s ideas about the role of food. The time had come to investigate the mechanisms underlying life history variation. A landmark paper that created focus on these underlying mechanisms was “The prudent parent: energetic adjustments in avian breeding” (Drent and Daan 1980). A famous part of this paper discusses the optimal workload of individual parents that care for their young:

We will argue that the working capacity of the parents is limited (in proximate fashion) by physiological constraints defining a sustained work level in metabolic terms. In some situations parent birds may ignore this physiological warning level, but the penalty will be a loss of condition which will in turn entail increased mortality. At this point much of the evidence is circumstantial… (Drent and Daan 1980).

In other words, Drent and Daan (1980) suggested that there are limits to how hard an individual should work for its offspring. Working too hard would negatively affect body condition, although these authors at that time did not specify exactly what comprised body condition. The first step was to demonstrate that hard work really did increase mortality. A classic example is a brood size manipulation experiment with kestrels (*Falco tinnunculus*) that showed that individuals triggered to work harder, because they received more nestlings to raise, showed a serious drop in survival during the next winter (Daan et al. 1996). The study of the kestrels was unique in following the individual birds throughout the winter after the experimental breeding season and determining the exact timing of death as well. Such manipulations of brood size and reproductive effort became characteristic for the 1980s and 1990s (e.g., Dijkstra et al. 1990; Norris et al. 1994; Moreno et al. 1995; Sanz and Tinbergen 1999). These types of studies suggested that the “loss of condition” was really there.

The mechanistic explanation for the trade-off between reproduction and survival, i.e., the units in which to measure the loss of condition, started to receive attention too. Originally, the mechanistic explanation focused on food and energetics (Drent and Daan 1980; Kersten and Piersma 1987; Daan et al. 1996), in line with David Lack’s hypothesis about the primary role for food in reproduction. This interest in energetics led to the introduction of field energetics methodologies (e.g., Hails and Bryant 1979; Weathers and Nagy 1980; Weathers et al. 1984; Williams and Nagy 1984; Buttemer et al. 1986) into studies of life history ecology, in addition to the use of already available respirometry techniques to measure metabolism in the laboratory (Lasiewski and Dawson 1967; Hill 1972; Withers 1977). A decade later, in the late 1980s and early 1990s, other physiological phenomena increasingly received attention from ecologists too: stress endocrinology (Wingfield and Silverin 1986; Astheimer et al. 1989), oxidative damage and aging (von Schantz et al. 1999; Haussmann et al. 2002; Monaghan and Haussmann 2006), and immune function (Norris et al. 1994; Sheldon and Verhulst 1996; Deerenberg et al. 1997; Nordling et al. 1998; Lochmiller and Deerenberg 2000; Norris and Evans 2000). Integrating measures of these physiological systems into ecological studies further improved the possibilities to qualify and quantify the elusive concept of “body condition.” In the course of time, a key shift occurred: these physiological traits started to be viewed as life history, or pace of life, traits themselves instead of merely mechanistic explanations underlying classical life history phenomena such as the trade-off between survival and reproduction (Ricklefs and Wikelski 2002; Sandland and Minchella 2003; Tieleman et al. 2004).

## Immune function in a life history context: a mechanism mediating trade-offs?

In the 1990s, at the start of the discipline of eco-immunology, virtually nothing was known about variation in immune function of free-living wild animals. This included, for the sake of the current paper, a lack of knowledge of immunological variation in birds too. Information was largely restricted to veterinary-based knowledge of zoo animals and poultry. The interest in eco-immunology arose more or less simultaneously in studies of a variety of ecological and evolutionary phenomena. The common denominator among these studies was an interest in what makes up “body condition.” Because natural selection favors “good quality” individuals, it is obviously relevant to ask what good quality really is. This applies to whatever context or phenomenon is of interest, including traits subject to sexual selection (Hamilton and Zuk 1982; Folstad and Karter 1992), how behavior is mediated by brain and immune function (psychoneuroimmunology), population dynamics (Lochmiller 1996), costs underlying life history-trade-offs (Deerenberg et al. 1997; Nordling et al. 1998), and predictions of which species might be good invaders into new areas such as cities (invasion biology, Lee and Klasing 2004).

Zooming in on how immunology entered and affected the field of life history ecology and evolution, and particularly the understanding of trade-offs between reproduction and survival/self-maintenance, I can evaluate hypotheses at the level of species and individual (Lochmiller and Deerenberg 2000; Lee 2006). Comparisons among species inform us about macro-evolutionary patterns; (experimental) studies at the level of individuals provide insights into micro-evolutionary processes. At the species level, the general hypothesis is that species with high reproductive rates have low survival and concordant low investments in immune function (Lochmiller and Deerenberg 2000; Ricklefs and Wikelski 2002; Martin et al. 2004, 2006; Tieleman et al. 2005; Lee 2006; Lee et al. 2008; Matson et al. 2009b; Versteegh et al. 2012). The analogy for individuals is that those that put in more (in an experimental context, i.e., too much) effort to reproduce do so at the expense of their immune defenses with the consequence that their probability of survival decreases (Deerenberg et al. 1997; Lochmiller and Deerenberg 2000; Tieleman et al. 2008; Hegemann et al. 2013a). These general hypotheses are sometimes refined to specify differential responses of different arms of the immune system that may be subject to different selective pressures (Lochmiller and Deerenberg 2000; Lee and Klasing 2004). Although these hypotheses were clear, it was not at all straightforward to describe the state of immune defenses in wild birds in a way that is both ecologically relevant and physiologically sound. Therefore, testing these hypotheses was preceded or accompanied by the development of assays to quantify immune function in free-living animals (Box 1).

**Box 1:** Describing immune defenses, in a way that is ecologically relevant and physiologically sound

The vertebrate immune system is highly complex and at the inception of eco-immunology in the 1990s was best known from studies of humans and domesticated animals. Unfortunately, that knowledge could not be directly used in the ecological context of wild animals. Doctors and veterinarians focused on making sick animals better by fixing specific problems. Most ecological and evolutionary questions asked what it takes for animals to stay healthy, i.e., how they *prevent* problems. These ecological and evolutionary questions required assays that allowed comparisons among species and individuals and were suitable for non-model organisms. In addition, they preferably could be applied while animals functioned naturally in their natural habitats, with minimal capturing and (repeat) sampling. That meant that the field of ecological immunology had to develop its own set of tools.
In the early days, ecologists sought biomedical or veterinary advice and emphasized measurements of the acquired arm of the immune system, such as antibody defenses triggered by the injection of sheep red blood cells (e.g., Deerenberg et al. 1997; Lochmiller and Deerenberg 2000). In the past 10–15 years, attention has shifted to the innate, non-specific arm of the immune system to the components that together make up the first line of defense and are thought to be more relevant for ecological and evolutionary questions. (Lochmiller and Deerenberg 2000; Lee 2006; Millet et al. 2007). Although many authors have applied these assays, many are still being fine-tuned and further studied to improve interpretation (Matson et al. 2005, 2006b, 2009a, 2012a, b; Millet et al. 2007; Tieleman et al. 2010; van de Crommenacker et al. 2010; Horrocks et al. 2011b). Current practice is to use a combination of assays to try to capture multiple components of the immune system (Lee 2006; Millet et al. 2007; Buehler et al. 2011, 2012; Evans et al. 2017). Despite the progress in developing and interpreting ecologically relevant immune assays, the field of ecological immunology still has important steps to make to summarize the complexity of an individual’s or species’ immune system, for example, in a limited number of key axes, in a way that describes its ecological functioning. Future progress in ecological immunology will be greatly served by further development of the ecological toolbox and of the ecological interpretation of the various measures.

## Immune function in a life history context: among species

Empirical support for the hypothesis of high investment in reproduction coinciding with low investment in immunity and low survival at the species level is mixed in comparative studies of birds. Whereas some studies support components of the hypothesis (e.g., Martin et al. 2001; Tella et al. 2002; Tieleman et al. 2005; Lee et al. 2008; Pap et al. 2015), others do not find support (e.g., Horrocks et al. 2012a, 2015; Versteegh et al. 2012). Notably, these latter studies do find that another component of physiology, namely metabolic rate, varies according to predictions with life history. In addition, several studies contradict each other. For example, Tella et al. (2002) do not find the relationship between cell-mediated immunity and clutch size that Martin et al. (2001) reported a year earlier. Yet, Tella et al. (2002) do find a relationship between cell-mediated immunity and longevity. Although difficult to quantify without in-depth study, as a research community, we may need to be aware of publication bias, as current scientific practice favors attention for positive and significant findings while deemphasizing negative and non-significant results. Studies that find no correlation between, for example, reproductive effort and components of immunity should be considered just as important and informative as those studies that do find such relationships.

The difficulty in extracting a general pattern of covariation between immune function and pace of life, and also the difficulty in devising meta-analyses in order to obtain a general pattern, may arise for a number of reasons: First, there is no common approach in selecting the most appropriate trait(s) to characterize pace of life, and the outcomes of searches for correlations between immunity and pace of life depend on the pace of life trait that is included (e.g., Tella et al. 2002; Lee et al. 2008). Some studies relate immune function directly with measures of reproduction (especially clutch size) or survival (lifespan) (e.g., Tella et al. 2002; Horrocks et al. 2012a), but more often studies use proxies that are considered part of the pace of life syndrome and expected to indirectly correlate with reproduction and survival, such as incubation period, development time, or metabolic rate (e.g., Tieleman et al. 2005; Lee et al. 2008; Pap et al. 2015). Perhaps ideally, one would like to use the probability of recruitment of individual offspring into the breeding population and the probability of survival of an adult from one breeding season to the next (Ricklefs 1983; Tella et al. 2002). Alternatively, a multivariate approach including a comprehensive set of life history traits to capture pace of life might be helpful (see also Araya-Ajoy et al. 2018, topical collection on Pace-of-life syndromes).

The second reason for the difficulty in extracting a common picture of immune function and life history is that there is a plethora of indexes to qualify and quantify the immune system of wild animals, and the outcomes of searches for correlations with life history traits are not consistent among different immune indexes within a single study and within identical immune indexes among different studies. To add to the complexity, different immune indexes only sometimes covary with each other, and patterns of covariation are not consistent among species or populations and over time, emphasizing the overlapping and redundant nature of the immune system (Matson et al. 2006a; Versteegh et al. 2012; Buehler et al. 2012). Examples to illustrate these points include some early comparative studies that used the, then fashionable, cell-mediated immune function (PHA wing web swelling) and found contradictory results. Whereas Martin et al. (2001) found a positive association between cell-mediated immunity and clutch size, Tella et al. (2002) could not repeat this with an extended data set. Studies that are contradictory within themselves include for example Lee et al. (2008), who show how one immune index (natural antibodies) varies with one pace of life trait (incubation period) and not with another (clutch size), while another immune index (complement) shows exactly the opposite pattern (varying with clutch size, not incubation period). Studies like this, revealing as they are about complexity and variation, leave us with an unsatisfactory answer to the question of a general fit of immune function into the pace of life syndrome.

A third reason for the lack of general patterns between immune function and life history may be that comparative studies of immune function and life history used different sets of bird species. Because of their differences in environment and ecology, this then tends to obscure comparisons of results among studies (see also Sandland and Minchella 2003). Some studies, for example, are restricted to the Neotropics (Tieleman et al. 2005; Lee et al. 2008), others to temperate Europe (Pap et al. 2015), while again others include a mixture of species from a variety of continents (Martin et al. 2001; Tella et al. 2002; Horrocks et al. 2012a, 2015).

Finally, the absence of a clear trade-off between immune function and life history traits could result from variation in acquisition hiding variation in allocation of resources, a classical explanation for contradictory results in life history studies (van Noordwijk and de Jong 1986; Reznick et al. 2000; Araya-Ajoy et al. 2018, topical collection on Pace-of-life syndromes). This problem is especially pertinent at the level of among-individual variation within a population, when individuals with more resources can have higher investments in two traits that are assumed to be traded off against each other, than individuals with fewer resources. At the level of comparisons among species, when working with trait values averaged over multiple individuals, this acquisition-allocation problem is generally smaller (van Noordwijk and de Jong 1986; Araya-Ajoy et al. 2018, topical collection on Pace-of-life syndromes). However, only experimental studies could unequivocally expose life history trade-offs.

In light of these potential reasons for the lack of general patterns between immunity and life history, I find it telling that more integrative studies that included multiple immune indexes and multiple life history traits, while controlling for ecological variation by focusing only on closely-related species, found no relationships between immune function and pace of life (Ardia 2007; Horrocks et al. 2012a, 2015; Versteegh et al. 2012).

## Immune function in a life history context: experiments at the individual level

Empirical support from experiments to test the individual level hypothesis is also ambiguous. This hypothesis states that those individuals that are experimentally triggered to invest more effort in reproduction do so at the expense of their immune defenses with the consequence that their probability of survival declines. Trade-offs between reproduction and immune function are reported frequently in experimental studies (e.g., Deerenberg et al. 1997; Nordling et al. 1998; Knowles et al. 2009), as are trade-offs between survival and immune function (e.g., Hanssen et al. 2004; Møller and Saino 2004). However, studies also abound that manipulated work load yet found no consequences for immune function, or found effects on only some immune indexes and not on others, or found effects that depended on location and year (e.g., Ilmonen et al. 2002; Ardia 2005; Tieleman et al. 2008; Knowles et al. 2009; Hegemann et al. 2013a). Recent reviews addressing the more general question of the physiological costs of hard work during reproduction point out that there is substantial variation in workload capacity among individuals, which in turn contributes to the individual variation in responses to experimental manipulations, including those of the immune system (Williams 2012; Williams and Fowler 2015). In addition, the time lag between manipulation and effect is variable, while typically only a single time point is measured (Sandland and Minchella 2003; Hegemann et al. 2013a; Williams and Fowler 2015). Specifically, the changes in parental immune function resulting from manipulation during the first brood sometimes do not appear during the first brood, but during subsequent broods later in the breeding season, or in the following year (e.g., Hegemann et al. 2013a). This variation in time lag and the finding in some studies that experimental effects of manipulations of reproductive effort on immune function vary among years or locations (e.g., Sandland and Minchella 2003; Ardia 2007; Hegemann et al. 2013a; Williams and Fowler 2015) suggest that the ecological and/or environmental context may feed into the dynamics between reproduction and immunity.

A final puzzling and complicating factor when studying the individual’s immune responses to experimentally altered reproductive effort is the lack of covariation among immunological indexes that is reported in many studies (Ardia 2007; Tieleman et al. 2008, 2010; Buehler et al. 2012; Hegemann et al. 2012, 2013a; Versteegh et al. 2012; Pigeon et al. 2013). In addition, when covariation is reported among some immune indexes, this is often species or population-specific and not repeated in other studies (Versteegh et al. 2012). For example, hemolysis and hemagglutination covary in studies on stonechats (*Saxicola* sp.) (Versteegh et al. 2012), red knots (*Calidris canutus*) (Buehler et al. 2008a; Buehler et al. 2011), and several species of waterfowl (Matson et al. 2006a), but not in kestrels (Parejo and Silva 2009) and five shorebird species (Mendes et al. 2006). These findings could be highly influential in directing future thinking about immune function in an ecological context. Somehow, as a research community, we have not yet come to grips with the complexity provided by the redundancy and overlapping functions within the immune system and the fact that each immune system is shaped by the unique history of disease and antigenic encounters in the course of an individual’s life time. Including these notions in hypotheses about ecological immunity, however, would fit with the observation that individual animals can vary different components of their immune system independent from each other and adds extra scope for understanding individual variation in responses to work load demands.

## Immune function in a life history context: conclusions

Evaluating the comparative and experimental studies of immune function in the context of life history trade-offs, my first conclusion is that there is tremendous variation in immune parameter values, both among species and among individuals. This raises the interesting question of why there is so much variation? My second conclusion is that, despite support by some studies, in general, the variation in immune function does not line up smoothly with life history variation at the species level, nor consistently explain life history trade-offs in experiments targeted at the within-individual level. Studies that diverge from predictions about the links between immunity and life history tend to have included large environmental or ecological variation, either because of including a large biogeographical scope (e.g., Ardia 2007; Horrocks et al. 2012a, 2015; Versteegh et al. 2012, 2014) or multiple years within a single study site (Hegemann et al. 2012, 2013a; Pigeon et al. 2013). This finding leads to the hypothesis that environmental conditions are more important in shaping immune function of birds than life history variation. This hypothesis deserves testing in a broader taxonomic context than birds, spanning a wider variety in immune systems and life history traits than present in the avian clade. In a review on invertebrates, Sandland and Minchella (2003) reached a similar conclusion and recommendation. Studies in other taxonomic groups such as mammals (e.g., Archie et al. 2012; Brock et al. 2013; Flies et al. 2015) also suggest that environmental and socioecological conditions affect immune function and need to be taken into consideration when exploring the nature of links between immune function and life history.

In addition, to build on the foundation in understanding immune function as life history trait, laid by the among-species comparisons and the within individual trade-offs reviewed in this paper, it would be useful to more thoroughly investigate among-individual variation in immune function in a variety of study systems. Among-individual level variation in immunity has thus far received relatively limited attention and among-individual correlations are generally reported at the phenotypic level only. To increase understanding of level-specific trade-offs will require studies with much larger sample sizes than are available from previous ecophysiological work, and study designs that allow teasing apart within and among individual effects, both genetic and environmental (van de Pol and Wright 2009; Dingemanse et al. 2012). I expect that such studies will also benefit greatly from including measures of the environmental and ecological contexts to understand variation in immune function.

## Immune function in the context of environment

It has been known for a long time that environment impacts life histories. For example, clutch sizes are smaller in the tropics than in temperate areas (Moreau 1944; Ricklefs 1980), growth rates of chicks are higher in the arctic than at lower latitudes (e.g., Schekkerman et al. 2003), and metabolic rates are low in desert (e.g., Tieleman and Williams 2000; Tieleman et al. 2004) and tropical regions (e.g., Tieleman et al. 2006; Wiersma et al. 2007) compared with temperate zones. Also for individuals, there is substantial variation in reproduction and survival related to environmental conditions, for example, among seasons and years. It is therefore perhaps not surprising that hypotheses were put forward about how and why immune function, as an upcoming life history trait, varies with environmental conditions. For example, Møller and Erritzoe (1996) found that the size of bird immune organs is associated with nest type and particularly nest reuse. In addition, Piersma (1997) suggested that arctic and marine waders would combine fast growth and costly migrations with low immune investments in relatively resource rich and parasite poor environments, compared with lower-latitude freshwater shorebirds that would have high immune investments. Similarly, deserts were proposed to provide not only fewer resources but also to harbor fewer diseases than the tropics, selecting for a slow pace of life in combination with low instead of high investments in immunity (Tieleman 2005; Horrocks et al. 2011a). And finally, Matson (2006) tested whether island birds had reduced immune defenses to evaluate the hypothesis that islands harbor relatively few diseases compared with continents. Generalizing the various hypotheses about connections between specific environments and immunity, among species, immune investments were proposed to depend on resources available for competing life history functions (such as growth, migration, or reproduction), while the balance between immune function and these other functions may be shifted by the level of infection risk (Lochmiller and Deerenberg 2000). At the individual level, investments in immunity were hypothesized to be balanced against competing annual cycle activities (such as growth, migration, or reproduction) and adjusted to the risk of becoming sick.

Although not extensively studied, support for these hypotheses linking immunity with environment exists both at the species level and at the individual level. For example, at the species level, Horrocks et al. (2015) found that among 12 lark species representing 23 environments, immunity was not associated with life history, but when plotted along a gradient of environmental aridity, ranging from temperate environments to hyper-arid deserts, various innate immune traits decreased when environments were drier. This finding is congruent not only with the idea that fewer resources are available for allocation to immune function in drier environments, but also with the assumption that drier areas are less diverse in general, including when it comes to disease-causing agents (Tieleman 2005). Other studies also show differences in immune function among environments, at biogeographic scales ranging from large, such as latitudinal gradients, to small, including variation in land use by humans (e.g., Martin et al. 2004; Buehler et al. 2008b; Buehler et al. 2009c; Pigeon et al. 2013; Zylberberg et al. 2013; Gutierrez et al. 2017), but a comprehensive review remains to be constructed.

At the individual level, environmental variation occurs at different levels, for example, among seasons or years within individuals or with different behaviors within or among individuals. Seasonal variation in immune function is well documented albeit for only a limited number of bird species (Buehler et al. 2008a; Pap et al. 2010a, b; Hegemann et al. 2012, 2013b; Horrocks et al. 2012b; Versteegh et al. 2014). From these studies, the seasonal pattern of variation is studied in multiple years only in the skylarks (*Alauda arvensis*), and surprisingly, two consecutive years can show largely the same pattern but also substantially diverge in some seasons (Hegemann et al. 2012). This is the case for multiple immune indexes (complement-mediated lysis, natural antibodies, haptoglobin) and raises the question of what causes differences in immunity among years? Candidate environmental factors to consider include food/diet, temperature, and disease dynamics/risk of infection within a population. In general, factors such as food and temperature may be more predictable than disease dynamics, and the question arises what the relative importance is of variation in each of these factors to explain the observed variation in immune function. The individual experience with these environmental factors may also be influenced by an individual’s behavioral style, potentially explaining among-individual variation in immune function. Proactive styles have been hypothesized to be associated with either higher (Barber and Dingemanse 2010) or lower (Réale et al. 2010) investment in immunity. A recent study on superb fairy wrens (*Malurus cyaneus*) found weak support for the latter based on a decrease in one (natural antibodies) of four immune indexes with exploration score, whereas the other indexes did not vary with this exploration score (Jacques-Hamilton et al. 2017).

The relative importance of different environmental factors in shaping the immune function of birds, at ultimate and proximate levels, remains understudied, especially in the field. Studies in captivity suggest that resource balance cannot explain everything. Stonechats*,* kept in a common garden setting in captivity with ad libitum access to food and constant room temperatures during the entire year, displayed significant changes in immune function in the course of the year (Versteegh et al. 2014). The annual pattern differed among subspecies, with the three subspecies involved originating from different environments (Versteegh et al. 2014). Experimentally modifying the annual pattern of temperature that the captive stonechats experienced into a dynamic pattern that resembled their natural situation more closely did not change their annual pattern of immune function (Versteegh et al. 2014), a finding corroborating an earlier study on red knots (Buehler et al. 2008b). Likewise, altering day length and food availability had little impact on indexes of constitutive innate immunity (Buehler et al. 2009a, b), but Buehler et al. (2009a) found that the acute phase response of red knots in captivity was affected when birds faced food restriction.

Experimental manipulations of environmental factors to study effects on immunity in free-living birds are difficult to achieve and have not been performed. A “natural experiment” with skylarks in the Netherlands, however, suggests potential for causal environmental effects on immune function (Hegemann et al. 2015). The skylarks of the breeding population in the Drents-Friesche Wold are partial migrants, which mean that some individuals stay around the breeding site in the winter, whereas others migrate south, most likely to France and the Iberian Peninsula. Using stable isotope values in the larks’ claws to determine whether birds stayed or not, Hegemann et al. (2015) compared body condition of locally wintering birds and their migrant counterparts. Although careful interpretation is needed because these birds were not randomly assigned to either strategy, the findings are intriguing. Considering migrating skylarks only, individuals that did not survive until the following breeding season had the same complement-mediated lysis titer as individuals that did survive. For residents, this was different: non-surviving residents had lower lysis scores before the winter than surviving residents. Thus, lysis appeared to partially predict survival for resident birds (Hegemann et al. 2015). Within-individual changes in lysis scores for birds that survived the winter also pointed to the direction of an environmental effect on immunity, because migrant birds scored similarly on lysis in two consecutive breeding seasons, but resident birds had a lower lysis score after spending the winter in the Netherlands (Hegemann et al. 2015). Although it is unclear which factors drive this, environmental factors provide a possible explanation. It would be worthwhile to follow up with experimental studies manipulating environmental circumstances in the winter, also in light of the dramatic decline of skylarks in most of north-western Europe (in the Netherlands, over 95% in the past four decades), which has been attributed to a poor winter food situation (Donald et al. 2001; Pan-European Common Bird Monitoring Scheme, PECBMS 2009).

Returning to my quest to understand variation in immune function in wild birds, I can now conclude that among species in different environments, immunity substantially varies, albeit not along the lines predicted with life history variation. Also within individuals, in different seasons or years, clear changes in immune function are documented, but the environmental factors that are the main causative agents remain to be pinpointed. While temperature and day length—of importance for resource balance—do not seem to have major proximate effects, food availability has effects on some components of the immune system but not on others, despite their substantial variation.

## Shifting the eco-immunology paradigm to include not only life history but also environmental and ecological contexts

Based on the eco-immunological studies on birds during the past 25 years, I propose that it is time to shift the eco-immunology paradigm. Thus far, eco-immunological questions have been rooted in a life history theoretical framework, in the context of which immune function is usually considered a physiological currency used to pay “costs” (remember the loss of condition in terms of the prudent parent from Drent and Daan (1980)). As a costly mechanism, immune function could then mediate and explain the widely observed trade-offs between survival and reproduction (and other life history traits such as growth or migration) (but see Williams and Fowler (2015) for a critical review of the costs of reproduction). The fact that the data so far provide only ambiguous support, at best, for this role of immunity, may be caused by the inclination to continue to view immune function mainly as a costly affair. Obviously, immune systems have not evolved to be costly; their primary purpose is to protect.

To understand the tremendous variation in immune systems among wild birds, I suggest to shift the paradigm to include—much more explicitly—the protective benefits of the immune system. An important step forward to achieve this is to include the environmental and ecological context prominently in the hypotheses. Also when these hypotheses concern pace of life phenomena, it will be paramount to include these environmental and ecological contexts to understand both potential costs and benefits of immune function. This suggestion connects with Réale et al. (2010), who, in a different context, argue for understanding and exploiting ecological variation in space and time in order to comprehend the interactions of various pace of life elements. Thus far, in the limited number of eco-immunology studies that explicitly considered the environment, its role has been largely hypothetical and based on assumptions, rather than on bird-relevant data that qualify and quantify the immune protection resulting from and demanded by different environmental or ecological circumstances. The realization that we need to map the environmental factors that challenge and shape the immune system confronts us with an enormous challenge. It requires capturing a holistic view of the environmental factors shaping immunity. Although daunting, this is a typical field ecological challenge: to describe the environment from the perspective of the animal. This environmental parameter would be something akin to the concept of (standard) operative temperature that emerged in studies of animal energy balance in the 1970s and 1980s and that was developed in response to a similar need to capture multiple temperature-related environmental factors at once and from the animal’s perspective (Porter and Gates 1969; Bakken 1980; Bakken et al. 1981). (Standard) operative temperatures include the effect not only of air temperature, but also of radiation, convection, and conduction, which together determine the thermal environment experienced by the animal and are measured with species-specific copper models (Bakken 1980; Bakken et al. 1981). In the case of the immune system, Horrocks et al. (2011a) previously proposed to call the environmental variable of interest “immunobiotic pressure.” This concept recognizes that what challenges and shapes the immune system is not only just pathogens (i.e., micro-organisms that, unless defended against, will actively cause sickness and death), but also other elements, including commensal micro-organisms, that often coevolve with the host. The goodness of fit between the immune defenses of the animal and the immunobiotic pressure posed by the environment will provide information about the effective operative protection experienced by the animal under specified environmental conditions (Horrocks et al. 2011a).

## Future outlook: including a microbial perspective to understand immune function in an environmental and ecological context

Whether or not “immunobiotic pressure” is a viable concept depends on the ability to quantify this entity or to split it up into components that can be quantified. Several exciting developments provide optimism that determining the immunobiotic pressure experienced by animals is within reach. Studies of wildlife disease that formerly focused on single infectious diseases increasingly expand to include multiple infections and infection histories (e.g., Ezenwa and Jolles 2011; Nunn et al. 2014; Henrichs et al. 2016), recognizing the need for a more holistic approach. Researchers intending to capture the infectious disease component of immunobiotic pressure can make use of sentinel birds (Komar 2001; Fall et al. 2013; Chaintoutis et al. 2014). Sentinel birds, raised in controlled environments, for example naïve to specific infections or in contrast explicitly exposed to certain infectious agents at known times during their development or life, can subsequently be exposed to the environment of interest to measure its immunobiotic pressure.

Another exciting development towards including the biogeographic and temporal landscape of immunobiotic pressures in understanding of the physiology, behavior, and life history of animals is the work on microbiomes (McFall-Ngai et al. 2013; Kohl and Carey 2016; Evans et al. 2017). Microbial communities comprise a large, and probably the largest, component of immunobiotic pressure. A pioneering field study that tried to relate immune function of free-living birds with microbial pressure measured the free-floating microbes in air and on the surface of birds (Horrocks et al. 2012a, b). Air is a component of the environment that every terrestrial animal encounters continuously no matter where it lives, thereby providing an omnipresent subset of the microbial environment. Horrocks et al. (2012a) used an air-sampling machine designed for industrial applications, where a controlled air flow passes through a lid with holes over an agar plate, on which microbes can grow colonies. They sampled air in two desert environments, where they had established that immune indexes of larks were low, and in a temperate environment, where immunity values of larks were high. Applying three different types of agar, to select for three broad classes of microorganisms, temperate zone air consistently contained more microbes than desert air, in line with the hypothesized differences. Although this study was limited by the use of culturing techniques, technological developments within microbial ecology over the past decade, including the accessibility and affordability of next-generation sequencing, have made it possible to describe microbial communities in extreme detail and to start to understand their functional properties (Amann et al. 1995; Hugenholtz 2002).

Including microbes in studies of animal physiology, behavior and life history promise more than establishing a component of immunobiotic pressure and thereby providing environmental context. Microbes are everywhere, not only in the environment surrounding the animal, but also on and in the animal. This realization makes the distinction between animal and surroundings fade: animal and surroundings become a continuum (Ruiz-Rodriguez et al. 2014; Avena et al. 2016; Lemieux-Labonte et al. 2016; Goodenough et al. 2017; van Veelen et al. 2017). The microbial ecology of the environment presents an immunobiotic pressure within which the bird (including its associated microbes) operates (the immunobiome; Horrocks et al. 2011a). A bird’s immune system may be importantly shaped by the microbial communities in the environment that it faces (Buehler et al. 2009c; Horrocks et al. 2011a, 2012a; Evans et al. 2017). The microbial communities living in and on the animal appear to be involved in many of the animal’s life history traits, including metabolism, immune function, reproduction, and behavior (Kohl 2012; Theis et al. 2012; McFall-Ngai et al. 2013; Evans et al. 2017). This is also the reason why the biomedical world is currently so interested in the human microbiome; it substantially influences human health and disease (Huttenhower et al. 2012; Lozupone et al. 2012; Methe et al. 2012; Yatsunenko et al. 2012). The question for evolutionary ecologists interested in the pace of life phenomenon is how, and by how much, do microbes affect immunity, metabolism, and growth, as well as survival and reproduction? Some authors propose to replace the concept of animals by holobionts, i.e., the host plus its associated microbes that can adapt quickly through changes in the microbial partners (Zilber-Rosenberg and Rosenberg 2008; Bosch and McFall-Ngai 2011; McFall-Ngai et al. 2013; Bordenstein and Theis 2015; but see Moran and Sloan 2015; Douglas and Werren 2016). In any case, the microbial inhabitants of hosts may provide a source of phenotypic plasticity to the host, with which it can rapidly adjust to alterations in the environment and that hitherto has not been considered in studies of pace of life. Especially the understanding of the tremendous amount of variation in immune systems of free-living animals, uncovered in the past 25 years, may benefit from a microbial perspective: it contributes understanding of the health benefits of immune functioning, makes visible a component of the immunobiotic pressure experienced by the animal, and provides insights into the animal microbiome’s effect on health and disease.
